# Risky decision-making and affective features of impulse control disorders in Parkinson’s disease

**DOI:** 10.1007/s00702-017-1807-7

**Published:** 2017-11-08

**Authors:** Alice Martini, Simon J. Ellis, James A. Grange, Stefano Tamburin, Denise Dal Lago, Greta Vianello, Nicola M. J. Edelstyn

**Affiliations:** 1 School of Psychology, Keele University, Staffordshire, UK; 2 Neurology Department, University Hospital of North Midlands NHS Trust, Stoke-on-Trent, UK; 3 Department of Neurosciences, Biomedicine and Movement Sciences, University of Verona, Verona, Italy

**Keywords:** Parkinson’s disease, Impulse control disorders, Decision-making, Cognition, Affective factors, Mood

## Abstract

Impulse control disorders (ICDs) in Parkinson’s disease (PD) are considered dopaminergic treatment side effects. Cognitive and affective factors may increase the risk of ICD in PD. The aim is to investigate risky decision-making and associated cognitive processes in PD patients with ICDs within a four-stage conceptual framework. Relationship between ICDs and affective factors was explored. Thirteen PD patients with ICD (ICD+), 12 PD patients without ICD (ICD−), and 17 healthy controls were recruited. Overall risky decision-making and negative feedback effect were examined with the Balloon Analogue Risk Task (BART). A cognitive battery dissected decision-making processes according to the four-stage conceptual framework. Affective and motivational factors were measured. ANOVA showed no effect of group on overall risky decision-making. However, there was a group × feedback interaction [*F* (2, 39) = 3.31, *p* = 0.047]. ICD+, unlike ICD− and healthy controls, failed to reduce risky behaviour following negative feedback. A main effect of group was found for anxiety and depression [*F*(2, 38) = 8.31, *p* = 0.001], with higher symptoms in ICD+ vs. healthy controls. Groups did not differ in cognitive outcomes or affective and motivational metrics. ICD+ may show relatively preserved cognitive function, but reduced sensitivity to negative feedback during risky decision-making and higher symptoms of depression and anxiety.

**Electronic supplementary material:**

The online version of this article (10.1007/s00702-017-1807-7) contains supplementary material, which is available to authorized users.

## Introduction

Impulse control disorders (ICDs) are repeated and excessive hedonistic behaviours that interfere in major areas of life functioning (Evans et al. 2009). ICDs include the four major impulse control disorders (ICDs), namely, pathological gambling, hypersexuality, compulsive shopping, and binge eating. Other compulsive behaviours such as punding, hobbyism, walkabout, hoarding, and dopamine dysregulation syndrome are often reported (Weintraub et al. 2015). An estimated 13–35% of PD patients are reported to develop clinically relevant ICD (Callesen et al. 2014; Garcia-Ruiz et al. 2014; Joutsa et al. 2012; Weintraub et al. 2010). However, this is likely to be an underestimation of their real prevalence in PD, for a number of reasons. First, people experiencing ICDs are reluctant to report them to their clinician due to embarrassment and shame. Second, patient’s insight may lack, even when caregivers recognize ICD as problematic, and information from caregivers is lacking in some studies in PD. Third, diagnostic criteria for gambling, binge eating (American Psychiatric Association 2013), and compulsive shopping (McElroy et al. 1994) do not consider ICDs as a continuum of severity and score them as either present or absent. Therefore, risky behaviours that fall short of the threshold will not receive a diagnosis even when they may impact on quality of life, health behaviours, financial stability, and relationships of patients and their families. They are important issues, as unrecognized and/or untreated ICDs will increase both the direct and indirect costs of PD.

A widely held view suggests that ICDs are side effects of dopaminergic replacement therapy (DRT) prescribed to ameliorate the cardinal motor symptoms of PD (Voon et al. 2007, 2011a). According to the “overdose hypothesis”, the requisite dopaminergic state necessary to control motor symptoms has the potential to move the same patient away from their optimum for certain cognitive functions (Cools et al. 2001; Cools and Robbins 2004; Gotham et al. 1988; Rowe et al. 2008), including decision-making. According to this hypothesis, the relationship between the efficiency of neuronal activity and the state of dopaminergic modulation is represented by a Yerkes–Dodson inverted U-shaped curve with decision-making and cognitive function declining with deviation away from optimum dosage for motor symptoms, indicated by the centre of the curve. This model implies that DRT may both improve and impair risk-taking behaviour depending on baseline dopamine levels in the underlying mesolimbocortical circuitry.

In the largest cross-sectional study of ICD in PD reported to date, at least one ICD was present in 13.6% of the cohort, ICDs were more common in patients medicated with both a dopamine agonist (DA) and levodopa (l-Dopa) compared to those taking only a DA or l-Dopa and a number of other factors, including younger age, being unmarried, current smoking, and a family history of gambling problems independently increased the risk of ICD (Weintraub et al. 2010). This study, also supported by wider literature findings, suggests a more complex relationship that includes both DRT and non-DRT factors. Converging evidence suggests that psychological factors, including depression, anxiety, and apathy, may contribute to ICD in PD (Callesen et al. 2014; Joutsa et al. 2012; Leroi et al. 2012; O’Sullivan et al. 2011; Pineau et al. 2016; Pontieri et al. 2015; Pontone et al. 2006; Voon et al. 2011b; Wu et al. 2015). In addition, a recent meta-analysis found impairment of decision-making, abstraction ability/concept formation, set-shifting, and visuospatial/constructional abilities as cognitive risk factors for ICD (Santangelo et al. 2017).

According to Sinha et al.’s (2013) conceptual framework, decision-making comprises four dissociable stages: option generation, option selection, action initiation or inhibition, and learning. The generation of behavioural options relies, at least in part, on perceptual and attentional mechanisms. These options are then valued and compared based on features including predicted reward, punishment, effort required, time involved to outcome delivery, and probability of outcome. The selected option is then associated with the appropriate action, but there is a fail-safe mechanism, whereby such action can be inhibited if the wider context changes and the chosen option is no longer advantageous. The real outcomes of behaviours are compared with predicted ones. Such comparisons play a key role in learning and feeding back on option selection mechanisms. Cognitive processes underlying each of these stages may be modulated, to some extent, by dopamine, with either lower or higher dopamine levels leading toward apathy or impulsivity according to the previously described inverted U-shaped curve. Moreover, factors other than dopaminergic modulation such as depression or anxiety can also influence processes within each stage.

Sinha et al. (2013) suggest that abnormal dopaminergic modulation of any one or combination of the four stages increases ICD vulnerability. Preliminary evidence provides partial support for this view. PD patients with ICD show disrupted option generation, since they seek less information before making a decision (Djamshidian et al. 2012, 2014) and abnormal option selection, as demonstrated by increased temporal discounting (i.e., the preference for immediate smaller rewards than bigger ones in the future) (Housden et al. 2010; Voon et al. 2010b). On the other hand, action initiation and inhibitory control appear to be relatively spared in PD patients with ICD based on performance on the Stop Signal and the Stroop tasks (Claassen et al. 2015; Djamshidian et al. 2011; Ricciardi et al. 2017). Similarly, learning from rewards was reported to be spared in PD with ICD (Piray et al. 2014; Voon et al. 2010a), whereas the effect of negative feedback on learning appears equivocal, with studies reporting either impairment (Leplow et al. 2017; Piray et al. 2014), sparing (Djamshidian et al. 2010) or no difference (Claassen et al. 2011).

To offer more information on this still unclear topic, we explored risky decision-making in PD with the Balloon Analogue Risk Task (BART), and dissected the four stages of the underlying conceptual framework (Sinha et al. 2013) through a battery of cognitive tests. The BART is a risk-taking task-related reward decision-making, where choices are based on explicit probabilistic information (Lejuez et al. 2002). The BART was shown to be an ecologically valid and sensitive measure of risk-taking behaviour, positively correlating with both self-report impulsivity and real-world risky behaviours, such as number of sexual partners, alcohol, smoke, and drug use (Hopko et al. 2006; Lejuez et al. 2002, 2003a, b). In addition, the BART has good reliability properties. Risk-taking behaviour on the BART does not differ across days. In addition, test–retest correlations across session are robust (*r* = + 0.77) (White et al. 2008).

A secondary focus was the relationship between ICD and previously identified affective and motivational factors.

In relation with our primary aim, we predicted that the cognitive profile of PD patients with ICD would be marked by a risky decision-making deficiency on the BART. Furthermore, according to the decision-making’s framework (Sinha et al. 2013) and wider PD literature, ICD risky decision-making deficit may be related to impairments in option generation, option selection, and learning. The extent to which inhibitory control is involved remains uncertain, given that three studies report sparing of PD with ICD performance on the Stop Signal task and Stroop tasks relative to PD without ICD performance (Claassen et al. 2015; Djamshidian et al. 2011; Ricciardi et al. 2017).

In relation with the secondary aim, we predicted that the PD with ICD group would be characterized by increased levels of depression, anxiety, and impulsivity but not apathy.

## Materials and methods

### Participants

PD patients were identified from a secondary care outpatient PD clinic and recruited if they met the following inclusion criteria: (a) idiopathic PD diagnosed by the UK PD Society Brain Bank Clinical Diagnostic Criteria (1992); (b) mild-to-moderate PD, defined as Hoehn and Yahr (H–Y) score 1–3 in ON condition; (c) Mini Mental State Examination ≥ 25/30; (d) able to provide written informed consent; (e) age = 35–85 years; and (f) medicated with levodopa (l-Dopa), dopamine agonists (DAs; i.e., pramipexole, ropinirole, rotigotine, apomorphine), monoamine oxidase B (MAO) inhibitors (i.e., selegiline, rasagiline), catechol-*O*-methyltransferase (COMT) inhibitors (i.e., tolcapone, entacapone), and/or amantadine.

Exclusion criteria were: (a) co-morbidity for other neurological illnesses other than PD; (b) history of learning difficulty including dyslexia; (c) physical inability to attend or comply with treatment scheduling, such as upper limb amputation, or crippling degenerative arthritis; (d) active malignancy; (e) family history of PD; (f) psychosis; (g) incapacitating dyskinesia on a stable dose of l-Dopa; and (h) medicated with either a centrally acting anticholinergic or atypical antipsychotic agents.

A total of 13 PD patients were allocated to the ICD (ICD+ group) and 12 as not harbouring ICD (ICD− group) by a certified neurologist (SJE) using published diagnostic criteria for dopamine dysregulation syndrome in PD (Giovannoni et al. 2000), punding (Evans et al. 2004), gambling disorder and binge-eating disorder (American Psychiatric Association 2013), compulsive shopping (McElroy et al. 1994), and hypersexuality (Voon et al. 2006). All ICD+ patients experience ICD in the 3 months preceding the screening visit.

The ICD+ and ICD− groups did not differ in any of the following clinical characteristics: age at PD onset, disease duration, Unified Parkinson’s Disease Rating Scale (UPDRS) parts III and IV, H–Y score, DA agonist use, and l-Dopa equivalent daily dosage (mg) (Tomlinson et al. 2010). None of the patients showed clinically relevant motor fluctuations (e.g., wearing off, OFF states).

Seventeen healthy controls provided baseline data for the cognitive measures. The control group met the same inclusion/exclusion criteria, apart from those related to PD. The three groups (i.e., ICD+, ICD−, and controls) were matched for age, sex, and years in education and they did not differ in current levels of cognitive functioning according to the Cambridge Cognitive Examination, (CAMCOG) (Roth et al. 1986). Premorbid crystallized IQ, measured with the Wechsler Test of Adult Reading (WTAR) (Wechsler 2009a), was significantly higher in healthy controls, but comparable between the PD groups. Daytime sleepiness, assessed with the Epworth Sleepiness Scale (ESS) (Johns 1991), was significantly higher in the ICD+ vs. healthy control group. Moreover, the three groups did not differ for marital status, alcohol, and cigarette consumptions which are previously identified risk factors for ICDs in PD (Weintraub et al. 2010). Baseline data are reported in Table 1.

**Table 1:** Baseline data, socio-demographic, habits, and clinical characteristics of the study sample

Variables	ICD+ (*n* = 13)	ICD− (*n* = 12)	HC (*n* = 17)	*F*, *t*, *χ* ^2^ values	*p*
Age (years)	64.62 ± 7.6, 65	63.42 ± 10.96, 66	68.65 ± 6.76, 68	*F*(2, 39) = 1.589	0.217
Male, *n* (%)	11 (84.6%)	10 (83.3%)	10 (58.8%)		0.271^§|^
Education (years)	13.77 ± 2.92, 13	13.58 ± 3.15, 12	15.23 ± 3.25, 15	*F*(2, 39) = 1.266	0.293
ESS	13.42 ± 3.65	9.58 ± 4.38	7.53 ± 4.54	*F*(2, 38) = 6.767	**0.003** ^§^
WTAR	108.23 ± 9.83	101.92 ± 6.39	116.12 ± 7.15	*F*(2, 39) = 11.690	**0.0001** ^†^
CAMCOG	96.77 ± 3.27, 97	94.75 ± 6.61, 95.5	99.29 ± 2.62, 99	*F*(2, 39) = 3.095^a^	0.057
Marital status	10/0/1/1	9/0/2/1	10/2/5/0		0.409^§|^
Cigarettes	6.67 ± 20.15, 0	0	0	*F*(2, 38) = 1.605	0.214
Alcohol	6.96 ± 2.37, 5.5	10.46 ± 2.92, 6.5	4.76 ± 0.95, 4	*F*(2, 38) = 2.053	0.142
Family history					
Alcohol abuse	1	1	0		
ICD	0	0	0		
Drug abuse	0	0	0		
PD features					
Age at onset	56.38 ± 7.73	57.08 ± 13.42	NA	*t*(23) = 0.158	0.876
Duration	8 ± 4.06, 8	7.29 ± 7.51, 5.5	NA	*U*(23) = 58.5	0.286
UPDRS-III	17.31 ± 5.96	20.92 ± 9.03	NA	*t*(23) = 1.188	0.247
UPDRS-IV	3.69 ± 3.97	3.17 ± 1.99	NA	*t*(23) = − 0.413	0.683
H&Y	2.5 ± 0.35	2.41 ± 0.70	NA	*t*(23) = − 0.37	0.716
LEDD (mg)	552.85 ± 353.42, 450	451.42 ± 293.77, 355	NA	*U*(23) = 61.00	0.355
DAs use, *n* (%)	10 (76.9%)	7 (58.3%)	NA		0.411^§§^
ICD type					
Single ICD	3/1/1/3^b^	0	0		
Multiple ICD	5^c^	0	0		

Continuous variables are presented as mean ± standard deviation, median. Median is reported for variables that were not normally distributed

Significant *p* values (*p* < 0.05) are given in bold

*ICD+* patients with ICD, *ICD*− patients without ICD history, *HC* healthy controls, *Education* years of formal education, *ESS* Epworth sleepiness scale, *WTAR* Wechsler Test of Adult Reading, *CAMCOG* Cambridge Cognitive Examination (total score), *Marital status* Married/widowed/divorced/single, *Cigarettes* number of cigarettes/week, *Alcohol* units/week, *UPDRS-III* unified Parkinson’s disease rating scale part III (motor score), *UPDRS-IV* unified Parkinson’s disease rating scale part IV (complications of therapy), *H&Y* Hoehn–Yahr disease severity rating scale, *LEDD* levodopa equivalent daily dosage, *DAs* dopamine agonist, *NA* not available, *Single ICD* hypersexuality/punding/binge eating/compulsive shopping

^§|^Fisher–Freeman–Halton test

^§§^Two-tailed Fisher exact test

^§^Post-hoc comparison: HC < ICD+; HC = ICD−; ICD+ = ICD−

^**†**^Post-hoc comparison: HC > ICD+; HC > ICD−; ICD+= ICD−

^a^Log10 transformed data

^b^Hypersexuality: *n* = 3, binge eating: *n* = 1, compulsive shopping: *n* = 1, punding: *n* = 3

^c^Hypersexuality + punding + compulsive shopping: *n* = 2, hypersexuality + punding: *n* = 1, hypersexuality + compulsive shopping: *n* = 1 hypersexuality + binge eating: *n* = 1

The study was approved by the NRES Committee West Midlands and Keele University research ethic committee. No study procedure was initiated before written informed consent was obtained.

### Cognitive assessment

#### Risky decision-making

**Fig. 1:**
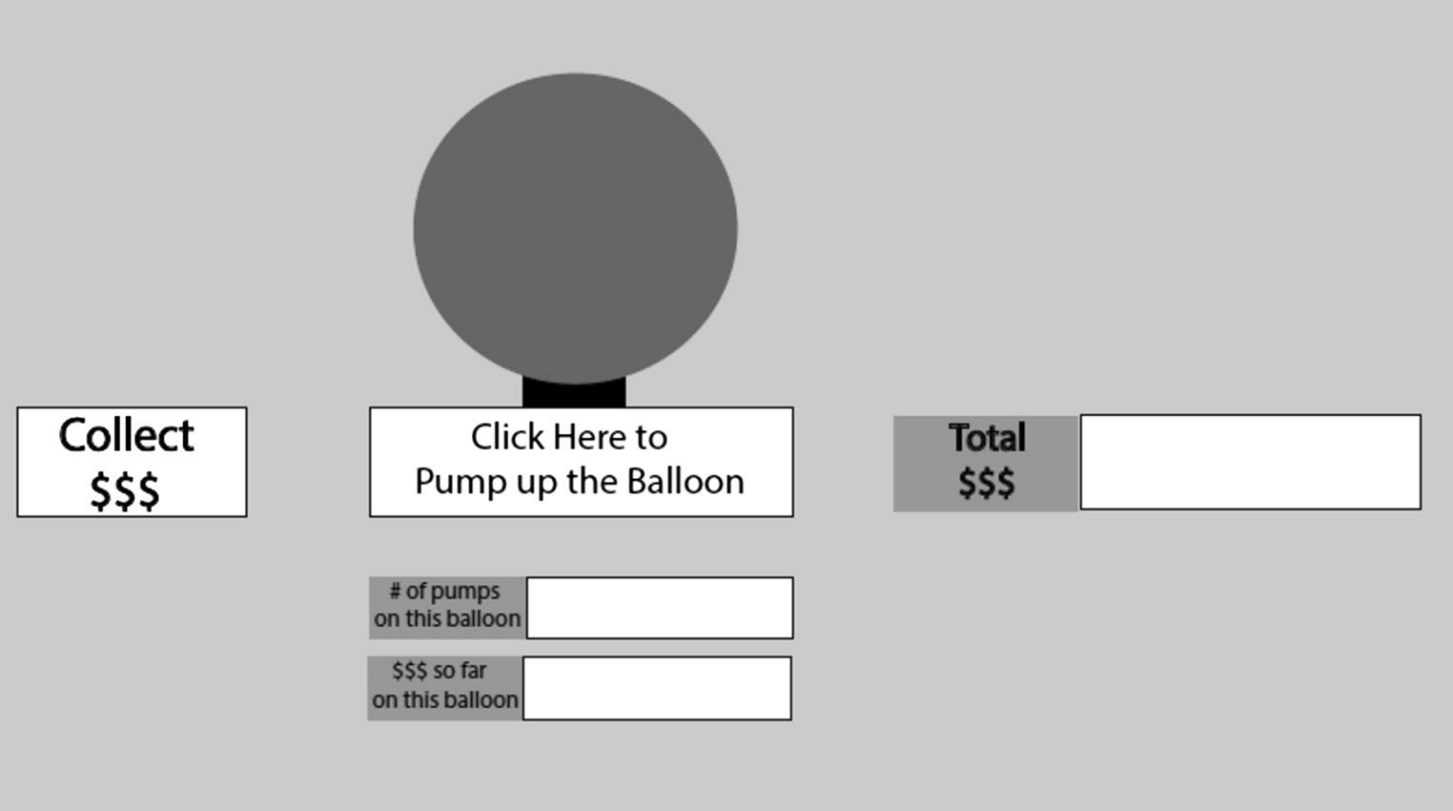
Balloon Analogue Risk Task (BART). The Balloon Analogue Risk Task is provided on a 17″ PC screen. Participants click with the mouse on the box labelled “Click Here to Pump up the Balloon” to inflate the balloon and earn money. Each pump earns 5 pence. Money can be collected by clicking on the box labelled “Collect $$$” and then transferred in the virtual bank named “Total $$$”. If the balloon is inflated too much, it could burst and the money is lost. Every time, a balloon bursts or money is collected, and a new balloon will appear. In total, there are 30 balloons. The number of pumps and money achieved for the current balloon is displayed in the boxes on the bottom of the screen

The Balloon Analogue Risk Task (BART) is a computerized task of risk-taking-related decision-making (Lejuez et al. 2002). An illustration of the task is provided in Fig. 1.

Participants sat in front of a 17″ PC screen and were instructed to use the mouse to incrementally inflate with each click a virtual balloon to gain virtual money. Each balloon pump earned 5 pence, but if a balloon was over-inflated and burst, the earnings were lost. To prevent losing money, participants had to stop inflating the balloon before it burst and click the “Collect $$$” button. The earnings were then transferred to a virtual bank, and a “slot machine reward sound” provided auditory feedback. The next trial commenced when a new balloon appeared following either a balloon burst or when earnings were banked. Each participant was asked to complete 30 trials.

Balloons were pre-programmed to burst at a “break point” in the range of 1–128 pumps. The probability that a balloon would burst was 1/128 for the first pump. If it did not explode, the probability for bursting was 1/127 for the second and so on. Therefore, the average break point was 64 pumps.

Two dependent variables were recorded. The first was the average number of pumps, where the balloon earnings were successfully cashed. For this outcome, the higher the number of pumps, the greater the risk-taking behaviour. The second was the average number of pumps for trials immediately preceding and immediately following a balloon burst (Claassen et al. 2011; Simioni et al. 2012). For this outcome, sensitivity for negative feedback was expressed as a lower number of pumps in trials that immediately followed a balloon burst compared to those that immediately preceded it. Participants were in ON condition when the BART was administered.

#### Cognitive battery

A cognitive battery was administered over two consecutive days, and at the same time of the day on each occasion. Each research session lasted 60–90 min. Patients did not show clinically relevant motor fluctuations. The researcher was blind to ICD status, but not to PD diagnosis.

The battery provided measures of divided attention (Test of Attentional Performance, TAP (Zimmermann and Fimm 1995), hit rate), and selective attention [Trail Making Test A, TMT-A (Reitan 1958), errors], executive function (CAMCOG, executive function subtest score), immediate and delayed story recall [Wechsler Memory Scale Logical memory subtest (Wechsler 2009b)], memory (CAMCOG, memory subtest score), verbal generation [The Hayling test—Section 1 (Burgess and Shallice 1997), scaled score], motor and verbal inhibitory control [Go/no-Go (Zimmermann and Fimm 1995), false alarms (i.e., reactions to a non-critical stimulus)]; The Hayling test—Section 2 [Burgess and Shallice 1997), scaled score], set-shifting [Trail Making Test B, TMT-B (Reitan 1958), errors], temporal discounting [the Kirby Delayed Discounting Questionnaire (Kirby et al. 1999) scored according to Myerson et al. (2014)], and rule detecting via feedback processing [the Brixton Test of Spatial Anticipation (Burgess and Shallice 1997), scaled score].

According to the four-stage decision-making framework (Sinha et al. 2013), the following outcomes provide estimates of (1) option generation: selective and divided attention, set-shifting, executive function, and verbal generation; (2) option selection: temporal discounting; (3) action initiation and inhibition: motor and verbal inhibitory control; and (4) learning: immediate and delayed story recall, memory, rule detecting via feedback processing, and negative feedback sensitivity from the BART.

### Affective and motivational factors assessment

Participants also completed subjective estimates of impulsiveness [Barratt Impulsiveness Questionnaire, BIS-11 (Patton et al. 1995)], anxiety and depression [Hospital Anxiety and Depression Scale, HADS (Zigmond and Snaith, 1983)], and apathy [Starkstein Apathy Scale (Starkstein et al. 1992)]. The questionnaires were completed at home between the two research sessions.

### Statistical analysis

Data were analysed with SPSS version 21.0 (SPSS, Chicago, IL, USA). For continuous variables, normality of distribution was explored with the Shapiro–Wilks test. For variables not normally distributed, log or squared root transformation was applied. Normally distributed transformed variables were analysed using parametric tests. When transformation did not solve normality, both parametric and nonparametric analyses were used to compare groups. Since the results were comparable for both types of analysis, results of parametric tests were considered, as they assure greater statistical power. Bonferroni was used as post-hoc test when ANOVA yielded significant differences between the three groups. Fisher’s exact test or Fisher–Freeman–Halton test was applied to categorical variables. *p* < 0.05 (two-tailed) was set as significance threshold for all the tests, except when Bonferroni correction for multiple comparisons was applied.

For BART, the average number of pumps on trials, where balloons were cashed, was compared between groups with a one-way ANOVA. Response to negative feedback was analysed with a 3 × 2 mixed-model ANOVA with the between-subjects factor group (ICD+, ICD−, and healthy controls) and the within-subject factor condition (pre-, post-burst).

A composite score of memory was calculated (using *z*-scores) from the CAMCOG memory subtest, immediate and delayed logical memory. Performance on each of the ten cognitive measures was analysed separately with a series of one-way ANOVAs, and Bonferroni corrected *p* < 0.005.

An exploratory Spearman correlational analysis examined the relationship between a single discrepancy score, derived from the difference in the number of pumps pre- and post-balloon burst, and the separate measures of selective and divided attention, memory, executive function, set-shifting, temporal discounting, inhibitory control, and rule detecting via feedback processing. A smaller discrepancy score reflects smaller changes in risk-taking behaviour after balloon burst and loss of virtual money.

## Results

### Risky decision-making

**Fig. 2:**
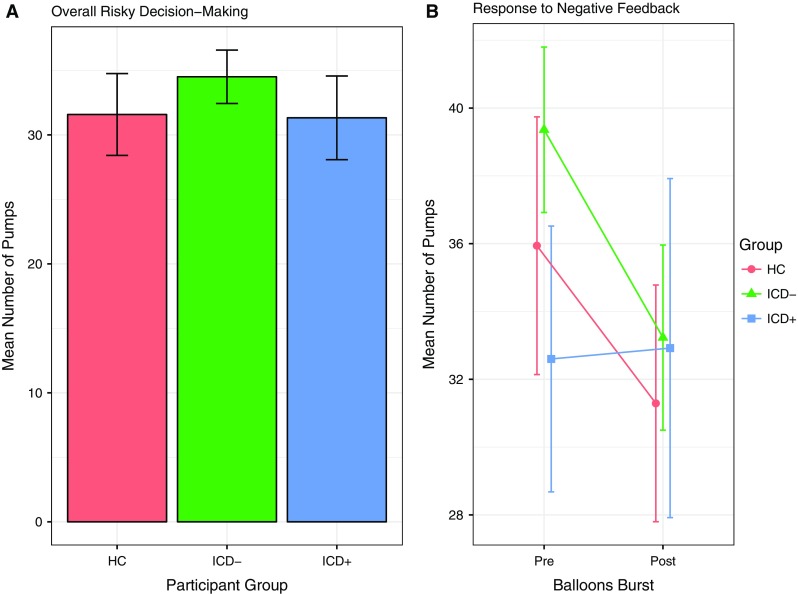
Performances in the BART. **a** Average number of pumps for cashed trials. The three groups did not differ in the average number of pumps, which reflects risk-taking behaviour. Higher scores represent riskier behaviours. Error bars represent the standard error of the mean. **b** Groups differ in the way they adjusted their behaviour after negative feedback. Negative feedback is expressed as the loss of money for trials in which a balloon burst. Both healthy controls (HC) and PD patients without ICD (ICD−) decreased the number of pumps after a negative feedback showing less risk-taking behaviour, whereas PD patients with ICD (ICD+) did not change their performance regardless of negative feedback. Error bars represent the standard error of the mean

The three groups did not differ in the average number of pumps on trials, where the balloon was cashed [ICD+ 31.32 ± 11.70; ICD− 34.51 ± 7.17; controls 35.58 ± 13.08; *F*(2, 39) = 0.31, *p* = 0.73; Table 2, Fig. 2a].

**Table 2:** Performances in the BART by groups and interactions

	ICD+ (*n* = 13)	ICD− (*n* = 12)	HC (*n* = 17)	*F* values	*p*
Average adjusted pumps	31.32 ± 11.70	34.51 ± 7.17	31.58 ± 13.08	*F*(2, 39) = 0.314	0.733
Negative feedback sensitivity	PRE: 32.6 ± 14.12 POST: 32.92 ± 18.01	PRE: 39.36 ± 8.47 POST: 33.23 ± 9.44	PRE: 35.94 ± 15.69 POST: 31.29 ± 14.38	Main effect of feedback *F*(1, 39) = 11.225	**0.002**
				Main effect of feedback × group *F*(2, 39) = 3.314	**0.002**
				Main effect of group *F*(2, 39) = 0.230	0.796

Data are presented as mean ± standard deviation

Significant *p* values (*p* < 0.05) are given in bold

*ICD+* Parkinson’s patients with ICD, *ICD−* Parkinson’s patients without ICD history, *HC* healthy controls, *SD* standard deviation, *Average adjusted pumps* average number of pumps in cashed balloons, *PRE* number of pumps for trails that immediately precede a balloon burst, *POST* number of pumps for trails that immediately follow a balloon burst

A comparison of the number of balloon pumps pre- and post-burst revealed no main effect of group [*F*(2, 39) = 0.23, *p* = 0.80]. However, there was a main effect of feedback [*F*(1, 39) = 11.23, *p* = 0.002], indicating that the mean number of pumps was significantly lower post- vs. pre-burst (pre 35.97 ± 2.11; post 32.48 ± 2.26). The group × feedback interaction was also significant [*F*(2, 39) = 3.31, *p* = 0.047], reflecting the observation that both the control and ICD− groups reduced their average number of pumps’ post-burst, whereas the ICD+ group showed no change (Fig. 2b). The reduction of pumps’ post-burst was significant for the control group [*t*(16) = 4.30, *p* < 0.001] and for the ICD− group [*t*(11) = 2.85, *p* = 0.02], but was not significant for the ICD+ group [*t*(12) = −0.14, *p* = 0.89].

### Cognitive battery

There were no significant differences between the three groups for any of the ten cognitive measures considered part of the four stages of decision-making using the strict Bonferroni corrected *p* < 0.005 (Table 3).

**Table 3:** Results of the cognitive test battery

Variables	ICD+ (*n* = 13)	ICD− (*n* = 12)	HC (*n* = 17)	*F* values	*p*
(1) Option generation stage					
TMT-A errors	0.31 ± 0.48, 0	0 ± 0, 0	0.18 ± 0.39, 0	*F*(2, 39) = 2.209	0.123
Divided attention hit rate	25.08 ± 3.75, 24	26.08 ± 5.82, 27	28.65 ± 2.50, 30	*F*(2, 39) = 3.056^a^	0.058
TMT-B^c^ errors	1.17 ± 1.53, 0.5	0.64 ± 0.67, 1	0.65 ± 0.93, 0	*F*(2, 37) = 0.967	0.390
Hayling Section 1 scaled score	5.46 ± 1.13, 6	4.75 ± 1.81, 6	5.65 ± 0.93, 6	*F*(2, 39) = 1.206^b^	0.319
CAMCOG EF	21.15 ± 2.97	20.83 ± 5.22	24.35 ± 2.37	*F*(2, 39) = 6.109^b^	0.008
(2) Option selection stage					
Kirby total^d^	0.47 ± 0.14	0.34 ± 0.12	0.49 ± 0.13	*F*(2, 38) = 4.658	0.016
(3) Action initiation and inhibition stage					
Go/no-Go false alarms	1.15 ± 1.28, 1	1.08 ± 2.27, 0	0.47 ± 0.72, 0	*F*(2, 39) = 0.988	0.381
Hayling Section 2 scaled score	5.31 ± 1.11	5.92 ± 0.67	6.06 ± 0.56	*F*(2, 39) = 3.519	0.039
(4) Learning stage					
Memory composite score	0.03 ± 0.83	− 0.54 ± 1.05	0.35 ± 0.64	*F*(2, 39) = 4.036	0.026
Brixton scaled score	3.46 ± 2.37, 2	3.5 ± 2.54, 2	5.65 ± 2.29, 6	*F*(2, 39) = 4.173	0.023

Data are presented as mean ± standard deviation, median. Median is reported for variables that were not normally distributed

*ICD+* Parkinson’s patients with ICD, *ICD*− Parkinson’s patients without ICD history, *HC* healthy controls, *TMT-A errors* Trial Making Test Part A number of errors, *TMT-B errors* Trial Making Test Part B number of errors, *Kirby total* Kirby Monetary choice questionnaire, *CAMCOG EF* Cambridge Cognitive Examination executive function subtest score

^a^Log10 transformed data

^b^Welch test (Levene test statistically significant)

^c^Two PD patients (one from the ICD+ and one from the ICD− groups) were excluded because not able to complete the TMT-B task

^d^One patient from the ICD+ group refused to complete the Kirby Questionnaire of Temporal Discounting

### Affective and motivational factor assessment

HADS total score was significantly different [*F*(2, 38) = 8.31, *p* = 0.001]. Bonferroni’s corrected post-hoc *t* tests revealed higher scores in the ICD+ group (3.68 ± 0.80) compared to controls (2.12 ± 1.08, *p* = 0.001), but no difference between the ICD+ and ICD− (3.05 ± 1.18) groups (*p* = 0.43) or between ICD− and controls (*p* = 0.068; Table 4).

**Table 4:** Affective and motivational characteristics by groups

	ICD+ (*n* = 12)	ICD− (*n* = 12)	HC (*n* = 17)	*F* values	*p*
HADS	14.17 ± 5.72, 15.5	10.58 ± 7.45, 9.5	5.59 ± 3.97, 5	*F*(2, 38) = 8.313^a^	**0.001** ^c^
HADS-A	8.25 ± 3.74	6.42 ± 5.26	4.12 ± 3.31	*F*(2, 38) = 3.683	**0.035** ^c^
HADS-D	5.92 ± 2.35, 6.5	4.17 ± 2.52, 3.5	1.47 ± 1.01, 1	*F*(2, 38) = 22.25^b^	**0.00001** ^d^
SAS	12.25 ± 4.75	11.66 ± 4.03	9.82 ± 2.94	*F*(2, 38) = 1.593	0.217
BIS-11	60.75 ± 10.28	58.33 ± 9.80	57.35 ± 10.59	*F*(2, 38) = 0.392	0.679

One participant from the ICD+ group refused to complete the questionnaires. Data are presented as mean ± standard deviation, median. Median is reported for variables that were not normally distributed

Significant *p* values (*p* < 0.05) are given in bold

*ICD+* Parkinson’s patients with ICD, *ICD−* Parkinson’s patients without ICD history, *HC* healthy controls, *SD* standard deviation, *HADS* Hospital Anxiety and Depression scale total score, *HADS-A* Hospital Anxiety and Depression scale anxiety sub scale, *HADS-D* Hospital Anxiety and Depression scale depression sub scale, *SAS* Starkstein Apathy scale, *BIS-11* Barratt Impulsiveness Scale total score

^a^Squared root transformed data

^b^Welch test (Levene test statistically significant)

^c^Post-hoc comparison: HC < ICD+; HC = ICD−; ICD+ = ICD−

^d^Post-hoc comparison: HC < ICD+; HC < ICD−; ICD+ = ICD−

Anxiety subscale score was significantly different [*F*(2, 38) = 3.68, *p* = 0.035]. ICD+ group showed higher scores (8.25 ± 3.74) than controls (4.12 ± 3.31, *p* = 0.032), but there was no difference between ICD+ and ICD− (6.42 ± 5.26) groups (*p* = 0.837) or between ICD− and controls (*p* = 0.432; Table 4). Depression subscale score was significantly different [*F*(2, 38) = 22.25, *p* = 0.00001]. Controls (1.47 ± 1.01) had lower scores than ICD+ (5.92 ± 2.35, *p* = 0.000002) and ICD− (4.17 ± 2.52, *p* = 0.002), but there was no difference between ICD+ and ICD− (*p* = 0.106; Table 4).

Scores for apathy and impulsivity from the Starkstein Apathy Scale and the BIS-11, respectively, did not differ between the three groups (Table 4).

### Exploratory correlation analysis

The BART discrepancy score, reflecting the difference in the number of pumps pre- and post-burst, negatively correlated with the Go/no-Go false alarms [*r*_*s*_(42) = − 0.336, *p* = 0.030]. This finding suggests that the more the sensitivity towards negative feedback, the fewer false alarms on the Go/no-Go task. No other correlations between BART discrepancy score and the cognitive outcomes were significant. Results for the full correlation matrix are presented in Table 5.

**Table 5:** Correlation matrix

Variable	TMT-A errors	TMT-B errors	Divided attention hit rate	Hayling Section 1	CAMCOG EF	Kirby total	Go/no-Go false alarms	Hayling Section 2	Brixton scaled score	Memory composite score
BART DS	*r* = 0.029 *p* = 0.855	*r* = 0.206 *p* = 0.203	*r* = 0.256 *p* = 0.101	*r* = − 0.209 *p* = 0.183	*r* = −0.096 *p* = 0.547	*r* = − 0.116 *p* = 0.469	*r* = − 0.336 ***p*** **=** **0.030**	*r* = − 0.72 *p* = 0.652	*r* = 0.191 *p* = 0.225	*r* = 0.080 *p* = 0.617
TMT-A errors		*r* = 0.365 ***p*** **=** **0.021**	*r* = − 0.191 *p* = 0.225	*r* = − 0.042 *p* = 0.792	*r* = − 0.214 *p* = 0.173	*r* = 0.170 *p* = 0.287	*r* = 0.225 *p* = 0.153	*r* = − 0.075 *p* = 0.637	*r* = − 0.056 *p* = 0.723	*r* = − 0.037 *p* = 0.817
TMT-B errors			*r* = − 0.256 *p* = 0.110	*r* = − 0.152 *p* = 0.349	*r* = − 0.399 ***p*** **=** **0.011**	*r* = − 0.021 *p* = 0.901	*r* = 0.401 ***p*** **=** **0.010**	*r* = 0.039 *p* = 0.811	*r* = − 0.13 *p* = 0.489	*r* = − 0.117 *p* = 0.473
DA hit rate				*r* = 0.139 *p* = 0.381	*r* = 0.543 ***p*** **=** **0.0002**	*r* = 0.151 *p* = 0.347	*r* = − 0.594 ***p*** **=** **0.00003**	*r* = 0.238 *p* = 0.129	*r* = 0.529 ***p*** **=** **0.0003**	*r* = 0.331 ***p*** **=** **0.032**
Hayling Section 1					*r* = 0.522 ***p*** **=** **0.0004**	*r* = 0.334 ***p*** **=** **0.033**	*r* = 0.025 *p* = 0.867	*r* = 0.233 *p* = 0.138	*r* = 0.182 *p* = 0.249	*r* = 0.464 ***p*** **=** **0.002**
CAMCOG EF						*r* = 0.297 *p* = 0.059	*r* = − 0.367 ***p*** **=** **0.017**	*r* = 0.256 *p* = 0.102	*r* = 0.565 ***p*** **=** **0.0001**	*r* = 0.578 ***p*** **=** **0.00006**
Kirby total							*r* = 0.127 *p* = 0.428	*r* = − 0.069 *p* = 0.666	*r* = 0.080 *p* = 0.621	*r* = 0.409 ***p*** **=** **0.008**
Go/no-Go false alarms								*r* = − 0.059 *p* = 0.710	*r* = − 0.565 ***p*** **=** **0.0001**	*r* = − 0.229 *p* = 0.114
Hayling Section 2									*r* = 0.154 *p* = 0.329	*r* = 0.293 *p* = 0.059
Brixton scaled score										*r* = 0.335 ***p*** **=** **0.030**

Significant *p* values (*p* < 0.05) are given in bold

*BART DS* Balloon Analogue Risk Task Discrepancy score, *TMT-A errors* Trial Making Test Part A number of errors, *TMT-B errors* Trial Making Test Part B number of errors, *CAMCOG* executive function: Cambridge Cognitive Examination executive function subtest score, *Kirby total* Kirby Monetary choice questionnaire.

## Discussion

This study investigated cognitive processes associated with risky decision-making in PD patients with and without ICD. The relationship between ICDs and previously identified affective factors was also explored.

In relation with our primary aim, we predicted that the cognitive profile of the ICD+ group would be marked by a risky decision-making deficit on the BART. The BART provides an ecologically valid estimate of risk-taking-related decision-making, and this is supported by correlations between performance on the BART and self-reported impulsivity and risk-taking behaviours in daily life, such as drugs abuse, alcohol abuse, and number of sexual partners (Hopko et al. 2006; Lejuez et al. 2002, 2003b). Our analyses, however, failed to support this prediction. When the three groups of participants were analysed together, risky decision-making performance did not significantly differ between ICD+, ICD−, and healthy controls.

These findings, however, can be reconciled with the previous reports of BART in PD with ICD (Claassen et al. 2011; Rao et al. 2010; Ricciardi et al. 2017). Risky decision-making on the BART successfully discriminated between ICD+ and ICD− in PD in relation with striatal BOLD response, where activation was diminished in ICD+ compared to ICD− (Rao et al. 2010), but abnormal brain activation in ICD+ was not mirrored by increased behavioural risk-taking. Furthermore, a medication withdrawal study showed that DRT increased risk-taking behaviour in PD reflecting an interaction between group and medication state, but there were no differences in risky decision-making behaviour between ICD+ vs. ICD− PD patients (Claassen et al. 2011).

These previous negative findings were suggested to depend on the use of a modified BART version, since a reduced range of possible pumps was found to be less sensitive to individual variability in task performance and, therefore, diminished the likelihood of detecting differences in risk-related constructs and self-report real-world behaviours (Lejuez et al. 2002).

The present BART data, which showed that risk-taking-related decision-making is spared in our cohort of ICD+ patients in comparison with ICD− and healthy control groups, together with results of the cognitive battery, offer an alternative explanation. According to Sinha et al.’s (2013) framework, decision-making is dependent on option generation, selection, action initiation and inhibition, and learning stages. Therefore, the likelihood of risky decision-making impairment will depend on the nature and extent of impairments within and across these four stages. We showed that cognitive processes underlying option generation, selection, and action initiation and inhibition in ICD+ were not different from other groups. However, we found an interaction between negative feedback and group, suggesting an impairment in the learning stage of decision-making process, that, despite being present, it is probably not by itself sufficient to affect overall risky decision-making performance on the BART. Interestingly, risky behaviour after negative feedback on the BART was significantly different in ICD+ patients, but performance on the cognitive tasks related to learning did not differ across groups. Therefore, learning appeared not to be abnormal in PD patients with ICD, unless reward is involved. Our findings are in keeping with the notion that learning and reward are supported by two separate cortico-striatal–thalamocortical circuits, i.e., associative and limbic circuits, respectively. The associative circuit links the dorsolateral prefrontal cortex with the dorsal caudate nucleus, and the limbic circuit links the ventral striatum with the ventral medial prefrontal cortex, orbitofrontal cortex, dorsal anterior cingulate cortex, amygdala, and hippocampus (Vriend et al. 2014b). We may speculate on a differential dopaminergic modulation of these two circuits in PD patients.

Abnormal response to negative feedback deficit was frequently reported in PD (Di Rosa et al. 2015; Frank et al. 2004; Jocham and Ullsperger, 2009; Volpato et al. 2016). Our study may extend this notion, suggesting that this impairment is more severe in ICD+ PD patients. According to Claassen et al. (2011), we scored negative feedback processing by comparing the number of pumps on the trials that immediately precede to those on trials immediately following a balloon bursts. Sensitivity toward negative feedback should result in decrease on risk-taking behaviour after a burst. Interestingly, we found reduced sensitivity to negative feedback in ICD+, while Claassen et al. (2011) failed to report differences between groups. As discussed above, we interpret these conflicting findings as due to differences on BART tasks used. Lower ranges of possible pumps could have decreased the variability across the overall score, as well as the trials considered for the negative feedback analysis.

According to event-related potential studies, negative feedbacks in BART are processed in the anterior cingulate cortex (ACC) (Schultz 1998, 2010), and are related to the reinforcement learning processes in the brain (Holroyd and Coles 2002) and the phasic dopaminergic dip signals. The ACC uses this signal to learn which action should be selected and executed. This provides a mechanism through which actions and events are linked to their outcomes with the goal of supporting decisions that maximize the opportunity to encounter reward in the future (Cockburn and Frank 2011).

Every time an outcome is better than expected or an unpredicted reward is received, DA neurons increase their phasic firing activity, and a positive prediction error is generated. In addition, every time an outcome is worse than expected or predicted reward is not delivered, there is a phasic dip in the DA neurons firings, and a negative prediction error is generated (Schultz 1998, 2010). DRT could prevent the DA neurons dip associated with negative feedback, as PD patients in OFF state are better when learning from negative than positive feedback, but they behave in the opposite way when ON medication (Frank et al. 2004). In both cases, not correctly generated prediction errors might lead toward abnormal links between actions and outcomes and impulsive behaviours may be facilitated.

In our study, the exploratory correlation analysis revealed a positive correlation between the BART discrepancy score and the number of false alarms in the Go/no-Go task supporting the importance of negative feedback processing for impulse control. The more participants decrease their risky behaviour after a negative feedback, and the fewer false alarms they make on the motor impulsivity task.

We also predicted that ICD+ would be characterized by increased impulsivity, and more symptoms of depression and anxiety but not apathy. Conversely, from our prediction, impulsivity levels were comparable between the three groups. BIS-11, which has not been validated in PD yet, may fail to differentiate between ICD− and ICD+ due to impulsivity presenting as a continuum of severity in PD despite the absence of clinical ICD. Comparable BIS-11 total score between ICD+ and ICD− is in keeping with the previous reports (Antonini et al. 2011).

We found depression and anxiety to be significantly higher in the ICD+ group than in healthy controls, but comparable between PD groups. The lack of differences between PD groups on the HADS score precludes any strong statement about the role of depression or anxiety in ICD. Nonetheless, the fact that ICD+ and healthy controls significantly differ on the HADS score may suggest one of the two not mutually exclusive explanations. The first is that higher depression and anxiety levels might increase vulnerability toward ICD in PD. Depression often precede PD diagnosis by several years (Ishihara and Brayne 2006; Tolosa et al. 2009) and both anxiety and depression symptoms are higher in PD than in the general population (Lieberman 2006; Prediger et al. 2012). In a subset of vulnerable PD patients, depression and anxiety symptoms could increase the risk of develop ICD as coping mechanisms. This hypothesis is supported by a retrospective study that showed higher baseline depression scores on drug naïve-PD patients who later developed ICD compared to PD patients who did not (Vriend et al. 2014a). Second, depression and anxiety could result as consequence of ICDs negative implications in patients and caregivers, as supported by reports of reduced quality of life in PD patients with ICD (Leroi et al. 2011; Phu et al. 2014).

## Limitations

There are several limitations in our study. First, this study has a small sample size and consequently low statistical power. Any interpretation of the results should be cautiously considered, since real differences between groups might fail to emerge when the number of participants is small. In the literature, underpowered studies investigating cognition in PD patients with ICD are frequent, as supported by a recently published meta-analysis in which 50% (17/34) of the studies reported have 20 or less participants in each group (Santangelo et al. 2017). Low recruitment rates are not surprising when experimental studies involve vulnerable older adults affected by a neurological condition such as PD which include motor, affective, and cognitive disabilities. Recruitment is even harder when studies focus on ICDs that are perceived with a sense of guiltiness, shame, and embarrassment. For these reasons, many PD patients with ICDs might be reluctant on taking part in research. Nonetheless, the small sample size and consequently reduced power are likely to affect our negative findings but not the results showing reduced sensitivity toward negative feedback in ICD+ vs. ICD− and healthy controls, or the higher depression levels in the ICD+ vs. healthy controls. Moreover, the small sample size prevented us to conduct separate analysis for ICD and punding, being the latter a probably separated but related phenomenon (Evans et al. 2009; Vriend et al. 2014b). Future and more powered studies are needed to further investigate cognitive and affective profiles of different types of ICDs.

Second, in the BART, not all the four components of the Sinha et al.’s (2013) framework could be measured, but only risk-taking behaviour after negative feedback, which we interpret as part of the learning stage. The framework’s components were assessed with the cognitive battery that was not significantly different between groups. The negative findings of the cognitive battery prevent any robust conclusion on the component(s) involved in the abnormal behaviour in PD patients with ICD. For example, ICD+ patients may not modify behaviour following negative feedback because of abnormal option selection mechanism, such as increased salience of rewarding stimuli. Further studies should include a task that can be broken down in the four different components of Sinha’s framework.

Third, healthy controls had significant higher scores in the WTAR, which is a measure of crystallized intelligence. This is unlikely to have affected our results, since participants showed comparable performances in CAMCOG, an extensive cognitive evaluation. Furthermore, since the comparable WTAR performances of the two PD groups, it is unlikely that ICD+’s abnormal responses to negative feedback were linked to crystallized intelligence.

Fourth, daytime sleepiness was significantly higher in ICD+ patients than healthy controls. However, it is unlikely that sleepiness levels could have affected our study results, since we found comparable outcomes in cognitive evaluation. Increased daytime sleepiness has already been reported in PD patients with ICD (O’Sullivan et al. 2011; Pontone et al. 2006; Scullin et al. 2013), probably because both represent side effects of DRT, especially DAs.

Fifth, ICD were diagnosed with a semi-structured interview following diagnostic criteria. This point might make direct comparison to the previous studies using QUIP-RS, a tool specifically validated in PD with ICD, difficult. Nonetheless, our more conservative approach assured that only patients with clinically relevant ICD were categorized as ICD+.

Sixth, UPDRS-III was administered at the screening visit only; therefore, the ON medication state was not controlled during all the assessment. Nonetheless, PD patients had low UPDRS-IV scores and they did not show any disabling motor fluctuation and/or OFF states.

Finally, other cognitive processes that have not been investigated in this study (e.g., incentive salience) may independently increase the risk to develop ICD or interact with negative feedback processing facilitating ICD development and/or maintenance.

## Conclusions

In conclusion, our findings suggest that abnormal negative feedback processing is a cognitive feature of ICD in PD which could account for impulsive behaviour in situations that involve both rewards and punishments. In addition, ICD in PD is associated with increased anxiety and depression than general population. Further studies are needed to understand whether abnormal negative feedback processing precedes ICD development, thereby constituting a premorbid vulnerability factor.

## Electronic supplementary material

Below is the link to the electronic supplementary material.

Dataset and codebook are available in the supplementary materials. (XLSX 49 kb)

## References

[CR1] American Psychiatric Association (2013). Diagnostic and statistical manual of mental disorders (DSM-5).

[CR2] Antonini A, Siri C, Santangelo G (2011). Impulsivity and compulsivity in drug-naïve patients with Parkinson’s disease. Mov Disord.

[CR3] Burgess PW, Shallice T (1997). The Hayling and Brixton tests.

[CR4] Callesen MB, Weintraub D, Damholdt MF, Møller A (2014). Impulsive and compulsive behaviors among Danish patients with Parkinson’s disease: prevalence, depression, and personality. Parkinsonism Relat Disord.

[CR5] Claassen DO, van den Wildenberg WPM, Ridderinkhof KR, Jessup CK, Harrison MB, Wooten GF, Wylie SA (2011). The risky business of dopamine agonists in Parkinson disease and impulse control disorders. Behav Neurosci.

[CR6] Claassen DO, van den Wildenberg WPM, Harrison MB, van Wouwe NC, Kanoff K, Neimat JS, Wylie SA (2015). Proficient motor impulse control in Parkinson disease patients with impulsive and compulsive behaviors. Pharmacol Biochem Behav.

[CR7] Cockburn J, Frank MJ (2011). Reinforcement learning, conflict monitoring, and cognitive control: an integrative model of cingulate-striatal interactions and the ERN. Neural Basis Motiv Cogn Control.

[CR8] Cools R, Robbins TW (2004). Chemistry of the adaptive mind. Philos Trans A Math Phys Eng Sci.

[CR9] Cools R, Barker RA, Sahakian BJ, Robbins TW (2001). Enhanced or impaired cognitive function in Parkinson’s disease as a function of dopaminergic medication and task demands. Cereb Cortex.

[CR10] Di Rosa E, Schiff S, Cagnolati F, Mapelli D (2015). Motivation-cognition interaction: how feedback processing changes in healthy ageing and in Parkinson’s disease. Aging Clin Exp Res.

[CR11] Djamshidian A, Jha A, O’Sullivan SS (2010). Risk and learning in impulsive and nonimpulsive patients with Parkinson’s disease. Mov Disord.

[CR12] Djamshidian A, O’Sullivan SS, Lees A, Averbeck BB (2011). Stroop test performance in impulsive and non impulsive patients with Parkinson’s disease. Parkinsonism Relat Disord.

[CR13] Djamshidian A, O’Sullivan SS, Sanotsky Y (2012). Decision making, impulsivity, and addictions: do Parkinson’s disease patients jump to conclusions?. Mov Disord.

[CR14] Djamshidian A, O’Sullivan SS, Lawrence AD (2014). Perceptual decision-making in patients with Parkinson’s disease. J Psychopharmacol.

[CR15] Evans AH, Katzenschlager R, Paviour D, O’Sullivan JD, Appel S, Lawrence AD, Lees AJ (2004). Punding in Parkinson’s disease: its relation to the dopamine dysregulation syndrome. Mov Disord.

[CR16] Evans AH, Strafella AP, Weintraub D, Stacy M (2009). Impulsive and compulsive behaviors in Parkinson’s disease. Mov Disord.

[CR17] Frank MJ, O’Reilly RC, Seeberger LC (2004). By carrot or by stick: cognitive reinforcement learning in parkinsonism. Science.

[CR18] Garcia-Ruiz PJ, Castrillo JCM, Alonso-Canovas A (2014). Impulse control disorder in patients with Parkinson’s disease under dopamine agonist therapy: a multicentre study. J Neurol Neurosurg Psychiatry.

[CR19] Giovannoni G, Sullivan JDO, Turner K, Manson AJ, Lees AJL (2000). Hedonistic homeostatic dysregulation in patients with Parkinson’ s disease on dopamine replacement therapies. J Neurol Neurosurg Psychiatry.

[CR20] Gotham AM, Brown RG, Marsden CD (1988). ’Frontal’ cognitive function in patients with Parkinson’s disease ‘on’ and ‘off’ levodopa. Brain.

[CR21] Holroyd CB, Coles MGH (2002). The neural basis of human error processing: reinforcement learning, dopamine, and the error-related negativity. Psychol Rev.

[CR22] Hopko DR, Lejuez CW, Daughters SB, Aklin WM, Osborne A, Simmons BL, Strong DR (2006). Construct validity of the balloon analogue risk task (BART): relationship with MDMA use by inner-city drug users in residential treatment. J Psychopathol Behav Assess.

[CR23] Housden CR, O’Sullivan SS, Joyce EM, Lees AJ, Roiser JP (2010). Intact reward learning but elevated delay discounting in Parkinson’s disease patients with impulsive-compulsive spectrum behaviors. Neuropsychopharmacology.

[CR24] Ishihara L, Brayne C (2006). A systematic review of depression and mental illness preceding Parkinson’s disease. Acta Neurol Scand.

[CR25] Jocham G, Ullsperger M (2009). Neuropharmacology of performance monitoring. Neurosci Biobehav Rev.

[CR26] Johns MW (1991). A new method for measuring daytime sleepiness: the Epworth sleepiness scale. Sleep.

[CR27] Joutsa J, Martikainen K, Vahlberg T, Voon V, Kaasinen V (2012). Impulse control disorders and depression in Finnish patients with Parkinson’s disease. Parkinsonism Relat Disord.

[CR28] Kirby KN, Petry NM, Bickel WK (1999). Heroin addicts have higher discount rates for delayed rewards than non-drug-using controls. J Exp Psychol Gen.

[CR29] Lejuez CW, Read JP, Kahler CW (2002). Evaluation of a behavioral measure of risk taking: the Balloon Analogue Risk Task (BART). J Exp Psychol Appl.

[CR30] Lejuez CW, Aklin WM, Jones HA, Richards JB, Strong DR, Kahler CW, Read JP (2003). The Balloon Analogue Risk Task (BART) differentiates smokers and nonsmokers. Exp Clin Psychopharmacol.

[CR31] Lejuez CW, Aklin WM, Zvolensky MJ, Pedulla CM (2003). Evaluation of the Balloon Analogue Risk Task (BART) as a predictor of adolescent real-world risk-taking behaviours. J Adolesc.

[CR32] Leplow B, Sepke M, Schönfeld R, Pohl J, Oelsner H, Latzko L, Ebersbach G (2017). Impaired learning of punishments in Parkinson’s disease with and without impulse control disorder. J Neural Transm.

[CR33] Leroi I, Ahearn DJ, Andrews M, McDonald KR, Byrne EJ, Burns A (2011). Behavioural disorders, disability and quality of life in Parkinson’s disease. Age Ageing.

[CR34] Leroi I, Andrews M, McDonald K, Harbishettar V, Elliott R, Byrne EJ, Burns A (2012). Apathy and impulse control disorders in Parkinson’s disease: a direct comparison. Parkinsonism Relat Disord.

[CR35] Lieberman A (2006). Depression in Parkinson’s disease—a review. Acta Neurol Scand.

[CR36] McElroy SL, Keck PE, Pope HG, Smith JM, Strakowski SM (1994). Compulsive buying: a report of 20 cases. J Clin Psychiatry.

[CR37] Myerson J, Baumann AA, Green L (2014). Discounting of delayed rewards: (A)theoretical interpretation of the Kirby questionnaire. Behav Process.

[CR38] O’Sullivan S, Loane CM, Lawrence AD, Evans AH, Piccini P, Lees AJ (2011). Sleep disturbance and impulsive-compulsive behaviours in Parkinson’ s disease. J Neurol Neurosurg Psychiatry.

[CR39] Patton JH, Stanford MS, Barratt ES (1995). Factor structure of the Barratt impulsiveness scale. J Clin Psychol.

[CR40] Phu AL, Xu Z, Brakoulias V (2014). Effect of impulse control disorders on disability and quality of life in Parkinson’s disease patients. J Clin Neurosci.

[CR41] Pineau F, Roze E, Lacomblez L, Bonnet AM, Vidailhet M, Czernecki V, Corvol JC (2016). Executive functioning and risk-taking behavior in Parkinson’s disease patients with impulse control disorders. J Neural Transm.

[CR42] Piray P, Zeighami Y, Bahrami F, Eissa AM, Hewedi DH, Moustafa AA (2014). Impulse control disorders in Parkinson’s disease are associated with dysfunction in stimulus valuation but not action valuation. J Neurosci.

[CR43] Pontieri FE, Assogna F, Pellicano C (2015). Sociodemographic, neuropsychiatric and cognitive characteristics of pathological gambling and impulse control disorders NOS in Parkinson’s disease. Eur Neuropsychopharmacol.

[CR44] Pontone G, Williams JR, Bassett SS, Marsh L (2006). Clinical features associated with impulse control disorders in Parkinson disease. Neurology.

[CR45] Prediger RDS, Matheus FC, Schwarzbold ML, Lima MM, Vital MA (2012). Anxiety in Parkinson’s disease: a critical review of experimental and clinical studies. Neuropharmacology.

[CR46] Rao H, Mamikonyan E, Detre JA, Siderowf AD, Stern MB, Potenza MN, Weintraub D (2010). Decreased ventral striatal activity with impulse control disorders in Parkinson’s disease. Mov Disord.

[CR47] Reitan R (1958). Validity of the Trail Making Test as an indicator of organic brain damage. Percep Mot Skills.

[CR48] Ricciardi L, Haggard P, de Boer L, Sorbera C, Stenner M, Morgante F, Edwards MJ (2017). Acting without being in control: Exploring volition in Parkinson’s disease with impulsive compulsive behaviours. Parkinsonism Relat Disord.

[CR49] Roth M, Tym E, Mountjoy CQ (1986). CAMDEX. A standardised instrument for the diagnosis of mental disorder in the elderly with special reference to the early detection of dementia. Br J Psychiatry.

[CR50] Rowe JB, Hughes L, Ghosh BCP (2008). Parkinson’s disease and dopaminergic therapy-differential effects on movement, reward and cognition. Brain.

[CR51] Santangelo G, Raimo S, Barone P (2017). The relationship between impulse control disorders and cognitive dysfunctions in Parkinson’s disease: a meta-analysis. Neurosci Biobehav Rev.

[CR52] Schultz W (1998). Predictive reward signal of dopamine neurons. J Neurophysiol.

[CR53] Schultz W (2010). Dopamine signals for reward value and risk: basic and recent data. Behav Brain Funct.

[CR54] Scullin MK, Sollinger AB, Land J (2013). Sleep and impulsivity in Parkinson’s disease. Parkinsonism Relat Disord.

[CR55] Simioni AC, Dagher A, Fellows LK (2012). Dissecting the effects of disease and treatment on impulsivity in Parkinson’s disease. J Int Neuropsychol Soc.

[CR56] Sinha N, Manohar S, Husain M (2013). Impulsivity and apathy in Parkinson’s disease. J Neuropsychol.

[CR57] Starkstein SE, Mayberg HS, Preziosi TJ, Andrezejewski P, Leiguarda R, Robinson RG (1992). Reliability, validity, and clinical correlates of apathy in Parkinson’s disease. J Neuropsychiatry Clin Neurosci.

[CR58] Tolosa E, Gaig C, Santamaría J, Compta Y (2009). Diagnosis and the premotor phase of Parkinson disease. Neurology.

[CR59] Tomlinson CL, Stowe R, Patel S, Rick C, Gray R, Clarke CE (2010). Systematic review of levodopa dose equivalency reporting in Parkinson’s disease. Mov Disord.

[CR60] Volpato C, Schiff S, Facchini S (2016). Dopaminergic medication modulates learning from feedback and error-related negativity in Parkinson’s disease: a pilot study. Front Behav Neurosci.

[CR61] Voon V, Hassan K, Zurowski M (2006). Prevalence of repetitive and reward-seeking behaviors in Parkinson disease. Neurology.

[CR62] Voon V, Potenza MN, Thomsen T (2007). Medication-related impulse control and repetitive behaviors in Parkinson disease. Curr Opin Neurol.

[CR63] Voon V, Pessiglione M, Brezing C, Gallea C, Fernandez HH, Dolan RJ, Hallett M (2010). Mechanisms underlying dopamine-mediated reward bias in compulsive behaviors. Neuron.

[CR64] Voon V, Reynolds B, Brezing C (2010). Impulsive choice and response in dopamine agonist-related impulse control behaviors. Psychopharmacology.

[CR65] Voon V, Mehta AR, Hallett M (2011). Impulse control disorders in Parkinson’s disease: recent advances. Curr Opin Neurol.

[CR66] Voon V, Sohr M, Lang AE (2011). Impulse control disorders in Parkinson disease: a multicenter case-control study. Ann Neurol.

[CR67] Vriend C, Nordbeck AH, Booij J (2014). Reduced dopamine transporter binding predates impulse control disorders in Parkinson’s disease. Mov Disord.

[CR68] Vriend C, Pattij T, van der Werf YD (2014). Depression and impulse control disorders in Parkinson’s disease: two sides of the same coin?. Neurosci Biobehav Rev.

[CR69] Wechsler D (2009). Test of premorbid functioning.

[CR70] Wechsler D (2009). Wechsler Memory Scale-Fourth Edition (WMS-IV).

[CR71] Weintraub D, Koester J, Potenza M (2010). Impulse control disorders in Parkinson disease: a cross-sectional study of 3090 patients. Arch Neurol.

[CR72] Weintraub D, David AS, Evans AH, Grant JE, Stacy M (2015). Clinical spectrum of impulse control disorders in Parkinson’s disease. Mov Disord.

[CR73] White TL, Lejuez CW, de Wit H (2008). Test–retest characteristics of the Balloon Analogue Risk Task (BART). Exp Clin Psychopharmacol.

[CR74] Wu K, Politis M, O’Sullivan SS (2015). Single versus multiple impulse control disorders in Parkinson’s disease: an (11)C-raclopride positron emission tomography study of reward cue-evoked striatal dopamine release. J Neurol.

[CR75] Zigmond AS, Snaith RP (1983). The Hospital Anxiety and Depression Scale. Acta Psychiatr Scand.

[CR76] Zimmermann P, Fimm B (1995). Test for attentional performance (TAP).

